# Size-Dependent Electroluminescence and Current-Voltage Measurements of Blue InGaN/GaN µLEDs down to the Submicron Scale

**DOI:** 10.3390/nano11040836

**Published:** 2021-03-25

**Authors:** Stefan Wolter, Hendrik Spende, Jan Gülink, Jana Hartmann, Hergo-Heinrich Wehmann, Andreas Waag, Andreas Lex, Adrian Avramescu, Hans-Jürgen Lugauer, Norwin von Malm, Jean-Jacques Drolet, Martin Strassburg

**Affiliations:** 1 Epitaxy Competence Center (ec^2^), Laboratory of Emerging Nanometrology (LENA) and Institute of Semiconductor Technology (IHT), Technische Universität Braunschweig, 38106 Braunschweig, Germany; h.spende@tu-braunschweig.de (H.S.); j.guelink@tu-braunschweig.de (J.G.); jana.hartmann@tu-braunschweig.de (J.H.); h.wehmann@tu-braunschweig.de (H.-H.W.); A.Lex@osram-os.com (A.L.); 2 Osram Opto Semiconductors GmbH, Leibnizstrasse 4, 98055 Regensburg, Germany; Adrian.Avramescu@osram-os.com (A.A.); Hans-Juergen.Lugauer@osram-os.com (H.-J.L.); Norwin.vonMalm@osram-os.com (N.v.M.); Jean-Jacques.Drolet@osram-os.com (J.-J.D.); Martin.Strassburg@osram-os.com (M.S.)

**Keywords:** GaN, µLED, size effect, electroluminescence, efficiency

## Abstract

Besides high-power light-emitting diodes (LEDs) with dimensions in the range of mm, micro-LEDs (μLEDs) are increasingly gaining interest today, motivated by the future applications of μLEDs in augmented reality displays or for nanometrology and sensor technology. A key aspect of this miniaturization is the influence of the structure size on the electrical and optical properties of μLEDs. Thus, in this article, investigations of the size dependence of the electro-optical properties of μLEDs, with diameters in the range of 20 to 0.65 μm, by current–voltage and electroluminescence measurements are described. The measurements indicated that with decreasing size leakage currents in the forward direction decrease. To take advantage of these benefits, the surface has to be treated properly, as otherwise sidewall damages induced by dry etching will impair the optical properties. A possible countermeasure is surface treatment with a potassium hydroxide based solution that can reduce such defects.

## 1. Introduction

In recent years the size dependent investigation of the electro-optical properties of micro-LEDs (μLEDs) has gained more and more interest [1,2,3,4,5,6,7]. While most of the present publications on this topic [1,3] cover only μLEDs with rather large cross-sectional areas, ranging between 500 × 500 μm^2^ and 10 × 10 μm^2^, an investigation of smaller μLEDs allows evaluating the potential of addressing submicron or even nano-LED structures. These structures are desirable because they can be used in the form of nano-LED arrays, which may open the way to controlling light at the nanoscale, e.g., for realization of a new type of super resolution microscopy [8]. In this sense, μLEDs with diameters in the range of 20 to 0.65 μm were investigated by electroluminescence (EL) and current-voltage (IV) measurements in this study.

The optical evaluation of μLEDs is based on external quantum efficiency (EQE), which can be used to characterize the interplay between the different recombination mechanisms, namely the Shockley–Read–Hall (SRH) recombination, the radiative recombination, and the Auger recombination. The EQE is related to the optical output power P and the injection current I as follows:

(1) $$\mathrm{EQE}=\frac{e\;P}{W_{\mathrm{Ph},\mathrm{av}}\;I}.$$

Here e is the electron charge and $W_{\mathrm{Ph},\mathrm{av}}$ is the photon energy averaged over the emission peak. Obviously, the optical power for a given current and hence the EQE can be determined by means of a calibrated optical detector, like an integrating sphere. A disadvantage of this method is that it requires fully processed μLEDs.

Nevertheless, under certain assumptions EQE curves in relative, non-quantitative units can be used to analyze μLEDs regarding their internal quantum efficiency (IQE). The theoretical background for this purpose provides the well-known ABC model which has been analyzed by many others before [9,10,11,12,13,14,15,16,17]. The ABC model is based on a rate equation, which connects the injection current I with the recombination coefficients A, B, and C, representing the non-radiative SRH recombination, the bimolecular radiative recombination, and the non-radiative Auger recombination, respectively [11].

(2) $$\frac{\eta_{\mathrm{inj}}\;I}{e\;V_{R}}=AN+BN^{2}+CN^{3}.$$

Here $\eta_{\mathrm{inj}}$ is the injection efficiency, $V_{R}$ is the recombination volume, and N is the charge carrier concentration. Under rather strict assumptions, like a balance between electron and hole concentration in the quantum well, an ideal injection efficiency and the independence of the recombination coefficients from the electron and hole concentration, the ABC model delivers the following expression [12,16]:

(3) $$\frac{{\mathrm{EQE}}_{\max}}{\mathrm{EQE}}=\frac{Q}{Q+2}+\frac{\sqrt{p}+1/\sqrt{p}}{Q+2},$$

where ${\mathrm{EQE}}_{\max}$ is the maximum external quantum efficiency achieved at a particular current density and hence carrier concentration, $p=P/P\left(\mathrm{EQE}={\mathrm{EQE}}_{\max}\right)$ is the normalized optical power, and $Q=B/\sqrt{AC}$ is the quality factor. As both ${\mathrm{EQE}}_{\max}/\mathrm{EQE}$ and p are normalized parameters, this equation can be analyzed even with a data set of output power vs. current density taken in arbitrary units. Hence, for a μLED that follows the ABC-model, a linear relationship between $\mathrm{EQE}/{\mathrm{EQE}}_{\max}$ and $\sqrt{p}+1/\sqrt{p}$ is expected, with the quality factor being the only fitting parameter. As the quality factor contains information about the different recombination mechanisms, it can be used to determine the maximum internal quantum efficiency ${\mathrm{IQE}}_{\max}$ [11,16]:

(4) $${\mathrm{IQE}}_{\max}=\frac{Q}{Q+2}.$$

Additionally, the ABC model allows the evaluation of the current dependence of the IQE [11]:

(5) $$\mathrm{IQE}=1-\frac{1-{\mathrm{IQE}}_{\max}}{2J}\;\left(1+\frac{\mathrm{IQE}\;J}{{\mathrm{IQE}}_{\max}\;J_{\max}}\right)\sqrt{\frac{\mathrm{IQE}\;J\;J_{\max}}{{\mathrm{IQE}}_{\max}}},$$

where J is the current density and $J_{\max}=J\left(\mathrm{IQE}={\mathrm{IQE}}_{\max}\right)$ is the current density at maximum IQE. $J_{\max}$ is not directly measurable, but can be approximated by the current density of maximum EQE if it is assumed that the light extraction efficiency (LEE) is independent from the current density. This implicit equation only depends on the parameters ${\mathrm{IQE}}_{\max}$ and $J_{\max}$, so that the IQE curve can be calculated without explicit knowledge of the rather complicated to determine recombination coefficients. Therefore, the ABC-model allows an in-depth analysis of the optical properties of μLEDs, although it relies on major assumptions, and the results should always be interpreted carefully.

While the extraction of ${\mathrm{IQE}}_{\max}$ and $J_{\max}$ is relatively straightforward, their size dependence is still controversial, as different tendencies have been published. While some contributions have indicated that with decreasing size the maximum quantum efficiency decreases and $J_{\max}$ increases [1,3,5], other publications showed that with the correct surface treatment both parameters remain more or less the same with decreasing size [2,7]. This topic is clearly highly relevant to the future of µLED display technology, when µLED diameters are reduced below 10 µm. Therefore, this topic was further analyzed here, based on a larger parameter range than previously published. One important aspect, explained in more detail later, should be pointed out here: reasonable information can only be drawn from high quality µLEDs showing a reasonable droop behavior. Only then the assumptions made above can be viewed as being fulfilled.

In addition to the analysis of quantum efficiency vs. size, other aspects turn out to be beneficial in µLEDs, like a reduction of leakage current paths in the forward direction with decreasing size. Furthermore, our analysis indicated that the light extraction efficiency increases with smaller size towards the top side, which is probably connected to light scattering at the etched sidewalls and the light guidance by waveguide modes. Additionally, it will be shown that ${\mathrm{IQE}}_{\max}$ and $J_{\max}$ are nearly size independent for the investigated sizes if the surface is treated carefully with a potassium hydroxide (KOH) based solution. These aspects suggest that submicron LED structures are feasible and that a further size reduction is possible and promising.

## 2. Materials and Methods

### 2.1. Samples and Fabrication

In this publication we investigated planar blue indium gallium nitride (InGaN)/gallium nitride (GaN) LEDs grown by metalorganic vapor phase epitaxy (MOVPE) on (0001)-sapphire substrates. These planar LED wafers were used to fabricate μLEDs, applying a top-down approach that combines photolithography and etching of the sidewalls. In the following a short summary of the µLED fabrication is presented. Details on LED growth can be found in a recent publication [18].

In a first step, palladium (Pd)/gold (Au) layers were deposited by electron beam evaporation on the p-GaN of the planar LEDs, which later serves as the p-contact. In order to obtain μLEDs with different sizes, a circularly shaped chromium (Cr) mask array with diameters between 20 and 0.8 μm was patterned onto the p-contact of the planar LED using photolithography and lift-off. After etching the whole structure down to the n-GaN by SF_6_/H_2_ inductively coupled plasma—reactive-ion etching (ICP-RIE), the Cr mask array was removed via commercially available chromium etchant and the resulting vertical μLEDs were treated with a 1 molar solution of potassium hydroxide for approximately 20 min at 80 °C. Finally, titanium (Ti)/Au n-contact pads were deposited by e-beam evaporation and patterned by photolithography between the μLEDs.

**Figure 1:**
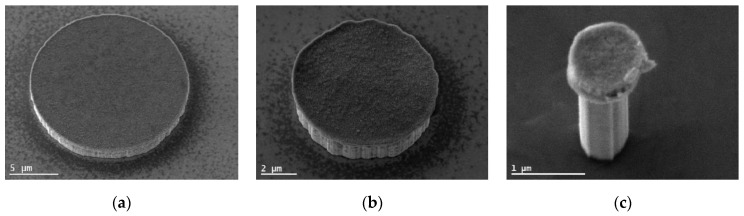
Secondary electron images in 30° tilted view of fabricated micro-LEDs (μLEDs) with a diameter of about (**a**) 18 μm, (**b**) 8 μm, and (**c**) 650 nm, respectively. The sidewalls are faceted due to the surface treatment with a KOH based solution, which has an anisotropic etching behavior, and partially due to the roughness of the Cr mask.

The KOH treatment removes surface damage that occurs during the ICP-RIE and smoothens the sidewalls [19], which should diminish the influence of sidewall defects. Moreover, it allows reducing the diameter of the μLEDs below even the limit imposed by the photolithography step; but in return, leaves the surface faceted due to its anisotropic etching behavior, as shown in Figure 1 for different μLED sizes. One possible measure to reduce the impact of surface facets is to reduce the initial size of the μLEDs before etching. This could be possible with the recently proposed nanosphere lift-off lithography, where the μLED size is mainly determined by the size of the used nanospheres [20].

### 2.2. Instrumentation

The characterization of μLEDs was performed in a Mira 3 GMH field emission—scanning electron microscope (FE-SEM; TESCAN GmbH, Dortmund, Germany) under high vacuum conditions. The FE-SEM system was equipped with MM3A piezo-driven micromanipulators (Kleindiek Nanotechnik GmbH, Reutlingen, Germany), which enabled precise positioning of tungsten metal tips on the corresponding contacts. These probe tips have a nominal tip radius of around 100 nm, so even the smallest structures with a diameter of around 650 nm can be contacted without problems. Furthermore, each probe tip was placed in a low-current measurement kit electro-mechanical tip holder that was connected to a Source Measure Unit 2636 (SMU; Keithley Instruments, Solon, OH, USA) via a triaxial cable to reduce electrical noise. While in this publication for current–voltage measurements a voltage was applied and the current was measured, the opposite was true for electroluminescence measurements. The emitted light was collected by a parabolic mirror, which was placed above the sample. The collected light was then coupled into a Mono CL 4 cathodoluminescence system (Gatan Inc., Pleasanton, CA, USA), where it passed through a Czerny–Turner monochromator and was directed to an iDus 420 BV CCD camera (Andor Technology Ltd., Belfast, Northern Ireland, UK), which recorded the EL spectrum.

### 2.3. Measurement Methods

Each μLED was investigated in a precisely predefined way. First, the diameter of the μLED was extracted from the secondary electron (SE) image. The μLED diameter serves as a parameter representing the size of the μLED and was used to calculate the current density, assuming a circular cross-section of the µLED. Second, the μLED was treated with a high current density of around 100 A/cm^2^ for about 1 min to ensure a homogenous p-contact, since the p-contact metal is only thermally deposited and therefore does not automatically form an alloy with the p-GaN. Third, the EL measurement was performed. For this purpose, the current values were selected in such a way that the current density of the maximum EQE can be estimated as accurately as possible. Typically, this means that the current density is swept between 0.01 A/cm^2^ and 100 A/cm^2^. Fourth, the current-voltage curve was recorded by sweeping the voltage from −10 to +6 V and measuring the current.

## 3. Results and Discussion

### 3.1. Electrical Properties

**Figure 2:**
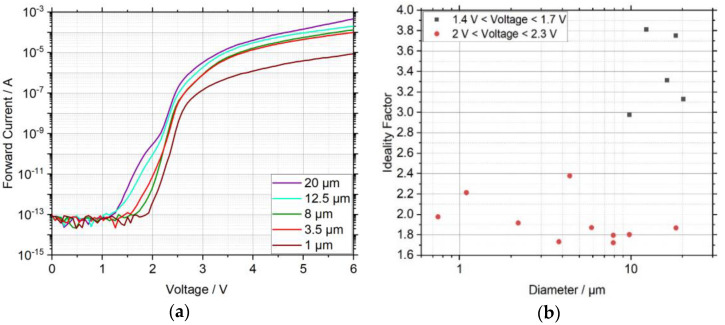
(**a**) Size-dependent IV curves of μLEDs with a diameter between 1 and 20 µm under forward-bias and (**b**) extracted ideality factors for different sizes and voltage ranges. The larger µLEDs show a premature rise of current for low forward voltages, which is accompanied by a large ideality factor. This indicates that for low voltages tunneling current dominates the carrier injection, while for moderate voltages diffusion–recombination current mainly contributes to carrier injection.

The size dependence of the electrical properties was determined by studying IV curves (Figure 2) for different μLED diameters. At very low voltages, the IV curves (Figure 2a) revealed a strong noise that corresponds to the current measurement limit of the low-current measurement kit electro-mechanical tip holder. The smaller structures showed a characteristic behavior: for low voltages the forward current increases exponentially with the forward voltage, and for voltages above about 2.5 V the exponential increase of current was limited by a series resistance, typically attributed to the contact resistances and the high resistivity of the p-GaN layer. In contrast to this, the IV curves of the larger structures rose prematurely at approx. 1 V, with a much smaller slope. The slope became steeper and similar to the slope of the IV curves of the smaller structures for voltages above 2 V, indicating that a change in current injection mechanisms occurred. The excess current at low voltages, which is particularly common for larger structures above 10 µm, cannot be attributed to a parallel resistance, since the reverse current for comparable reverse voltages was smaller by orders of magnitude. It can rather be explained by a parasitic parallel diode, or more precisely leakage current paths through the μLED that support the current flow preferably in the forward direction. As the forward current does not contain a component that scales linearly with the diameter, surface leakage currents do not seem to be a reasonable approach to describe the excess current. Even though the origin of this excess current may not be completely clear, it does not jeopardize the key conclusions drawn from the analysis of the small µLEDs.

A typical way to investigate the current injection mechanisms consists of fitting an exponential equation $I\left(V\right)=I_{0}\exp\left(\frac{V}{n_{\mathrm{ideal}}V_{T}}\right)$ to the measured data [21], where $I_{0}$ is a pre-exponential factor, $n_{\mathrm{ideal}}$ is the ideality factor, and $V_{T}$ is the thermal voltage that has a value of 25.86 mV at a temperature of 300 K. The extracted ideality factors for different sizes and for voltages between 1.4 V and 1.7 V, respectively 2.0 V and 2.3 V, are shown in Figure 2b. Regarding the leakage current, the ideality factor lies between 3 and 4, which is outside the range of 1 to 2 predicted by the simple theory and therefore indicates that additional current paths dominate the carrier injection, being interpreted as tunneling current in [22]. This is in agreement to similar publications on this topic [23,24,25], which showed that the current leakage at low voltages could be caused by carrier tunneling to deep level traps within the space-charge region followed by a recombination step. For the second region, the ideality factor is always near the value 2, or even slightly below, indicating that diffusion–recombination current dominates. As a contribution to tunneling current in the forward direction, deep level states associated with threading dislocations (TDs) were supposed by other working groups [23,24]. Accordingly, the weaker tunneling behavior of the smaller structures is reasonable because the number of TDs per µLED decreases with the size. For the investigated LEDs the density of TDs was typically at about 10^8^ cm^−2^, so for the smaller µLEDs the expected number of TDs per average lies within the range of one or even zero. Nevertheless, since the current for low voltages, and thus the tunneling region, could not be resolved for the smaller structures, it cannot be excluded that tunneling currents also dominate the carrier injection in this region.

### 3.2. Optical Properties

**Figure 3:**
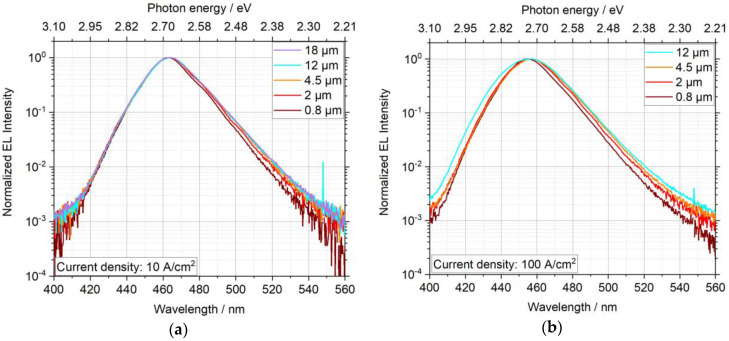
Size-dependent normalized EL spectra at a current density of (**a**) 10 A/cm^2^ and (**b**) 100 A/cm^2^ for μLEDs with a diameter between 0.8 and 18 µm. A semilogarithmic scaling was selected to show the small changes in the curve shape for different sizes. At a current density of 100 A/cm^2^, the emitted light of the µLED with a diameter of 18 µm saturated the CCD camera so that the spectrum was omitted.

The normalized EL spectra for different sizes are plotted in Figure 3 for two different current densities. Obviously, the shape of the spectra changes not only with current density but also with size of the µLEDs. At low current densities of 10 A/cm^2^ (Figure 3a), the shorter wavelength regime of the spectrum was almost identical for all µLEDs, whereas in the longer wavelength regime of the spectrum the intensity declines faster with increasing wavelength as the size decreases. At higher current densities of 100 A/cm^2^ (Figure 3b) this decline at longer wavelengths was even more pronounced and the same phenomenon was also visible at shorter wavelengths. This behavior was mainly attributed to a small shift in the peak of photon energy and to a broadening of the spectrum with current density. The broadening could be associated with local heating of the sample during the measurement, because the full width at half maximum (FWHM) increased with temperature due to the influence of the carrier distribution function. In this sense, a smaller temperature rise, and thus better heat dissipation, for smaller µLEDs might be caused by surface heat radiation that scales with the surface to volume ratio. Furthermore, the inhomogeneity of indium content within the four quantum wells is expected to be lower for smaller structures, which in turn also means that a lower FWHM is expected with smaller structure sizes.

**Figure 4:**
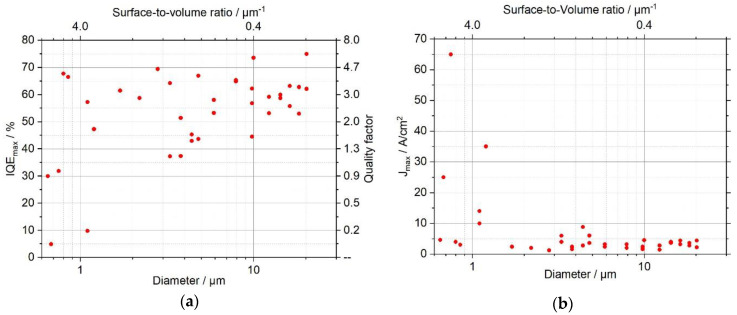
Size-dependent (**a**) maximum internal quantum efficiency ${\mathrm{IQE}}_{\max}$ and (**b**) current density at the maximum internal quantum efficiency $J_{\max}$ derived from the EL spectra by using the ABC model. With decreasing µLED diameter, ${\mathrm{IQE}}_{\max}$ and quality factor Q decrease, while $J_{\max}$ increases.

Using the methods described in the introduction, the EL spectra measured for different current densities and µLED sizes were evaluated to determine the size-dependent behavior of ${\mathrm{IQE}}_{\max}$ and $J_{\max}$, which are shown in Figure 4a,b, respectively. For all values in Figure 4 the ABC model with the underlying assumptions seems to be a reasonable approach, as the measured data does not significantly deviate from the linear fit according to Equation (3). While ${\mathrm{IQE}}_{\max}$ as well as $J_{\max}$ varied only moderately for larger structures (d≳2 μm), both varied strongly for smaller structures (d≲2 μm). For larger structures ${\mathrm{IQE}}_{\max}$ was between 50 and 70%, and $J_{\max}$ lies within the range of 1 to 9 A/cm^2^, whereas at least for some of the smaller structures ${\mathrm{IQE}}_{\max}$ decreased to about 5 to 30%, and $J_{\max}$ increased to approximately 25 to 65 A/cm^2^. With regard to the occurring recombination mechanisms, possible explanations for an increase of $J_{\max}$ with decreasing size are an increase in SRH recombination, an increase in radiative recombination, or a decrease in Auger recombination. Only the increase in SRH recombination is consistent with the observed decrease in ${\mathrm{IQE}}_{\max}$ for small LEDs. An increase in SRH recombination with decreasing size most likely corresponds to the fact that the influence of surface states or sidewall defects acting as SRH recombination centers increases, since both scale with the surface-to-volume ratio. Both contributions are associated with dangling bonds occurring at the surface, whereas their physical origin is different. While surface states describe dangling bonds that occur because the crystal periodicity is not continued at the surface, sidewall defects are caused by the breaking of bonds due to ion bombardment during ICP etching. Therefore, the latter can reach even further into the semiconductor.

It is interesting to note that even with small sizes some µLEDs still show the typical values for ${\mathrm{IQE}}_{\max}$ and $J_{\max}$, indicating that the performance of µLEDs depends strongly on the manufacturing process. It is reasonable to assume a correlation with the density of defects occurring during ion etching. It is well known that KOH wet etching can substantially reduce such defects. Wong et al. showed that the treatment of the µLED with KOH recovers their performance by removing leakage current paths at the sidewall [7]. In addition, the surface structure is modified due to the anisotropic etching behavior of KOH for GaN, as shown in Figure 1. Therefore, the decrease of ${\mathrm{IQE}}_{\max}$ and increase of $J_{\max}$ strongly depends on how much of the surface volume, which has been prone to ion bombardment, was removed during wet etching and how the surface shape was changed. These processes are not yet well under control and differ significantly even for µLEDs of identical size. Comparing the influence of surface states and sidewall defects, the latter is considered to be the main contribution to the observed phenomenon. This can be traced back to the strong increase of surface recombination velocity by sidewall defects caused by dry etching, as observed by Boroditsky et al. [26]. To sum up, by carefully treating the surface with KOH, the internal quantum efficiency of the µLED, represented by ${\mathrm{IQE}}_{\max}$ and $J_{\max}$ according to Equation (5), can recover very well for all investigated sizes. We conclude from our results that there is obviously no intrinsic mechanism that automatically reduces the ${\mathrm{IQE}}_{\max}$ in µLEDs as the dimensions are reduced down to 1 µm and below.

**Figure 5:**
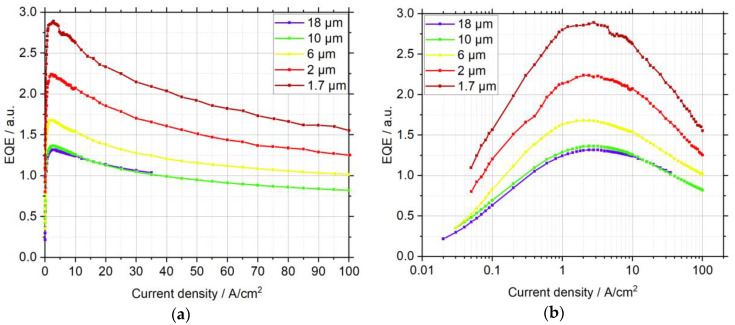
Size-dependent external quantum efficiency curves in arbitrary units displayed with (**a**) linear, and (**b**) semilogarithmic, scaling. The measured external quantum efficiency (EQE) increases with decreasing size, which can be explained by an increase of light extraction at the sidewall.

From this it follows that an analysis of the external quantum efficiency is quite interesting, because a size-dependent investigation of μLEDs with similar IQE curves reveals changes in the light extraction efficiency with size. In this spirit, Figure 5 shows the size-dependent EQE for µLEDs which had similar values for ${\mathrm{IQE}}_{\max}$ and $J_{\max}$. As the measured EQE contains only the light that is coupled into the spectrograph through the parabolic mirror above the sample, an evaluation of the light extraction efficiency in absolute numbers is not possible. Nevertheless, Figure 5 shows that the EQE curves in arbitrary units merge for small, as well as for high, current densities and increase with decreasing size, indicating that a change in the radiation characteristics occurs. For smaller structures light seems to be preferably coupled out to the top, which is in good agreement with the results of Choi et al., who suggested that smaller μLEDs have better top and sidewall light extraction, because the light is scattered from the etched sidewall and can propagate in resonant cavity modes [27].

## 4. Conclusions

A thorough analysis of current-voltage and electroluminescence measurements showed that the electro-optical properties of blue InGaN/GaN µLEDs change significantly with size in a range of diameters from 0.65 to 20 µm. The IV curves indicated that smaller µLEDs show less current leakage in the forward direction, whereas this current leakage is strong for the larger µLEDs, and can probably be attributed to the presence of threading dislocations. From the evaluated EL spectra it follows that the µLEDs had an improved light emission towards the top side. Furthermore, using the ABC model, a decrease of ${\mathrm{IQE}}_{\max}$ and an increase of $J_{\max}$ with decreasing size was observed, which can mainly be explained by an increased SRH recombination at sidewall defects induced by ICP-RIE. The measurements also showed that a careful treatment of the surface with KOH improves the IQE and could possibly recover the optical properties of the µLEDs after dry etching. The key message is that no intrinsic mechanism was observed which automatically decreases the IQE of µLEDs for diameters below 1 µm. In particular, this also means that an investigation of even smaller structures will be of interest in the future, since the influence of surface states was low for the currently investigated structures and therefore of no concern. This should at least change when the size of the µLED lies in the range of the depletion region induced by the surface states, where surface passivation becomes necessary to maintain a current path within the LED.

## Data Availability

Data available in a publicly accessible repository. The data presented in this study are openly available in the repository of the Technische Universität Braunschweig at doi:10.24355/dbbs.084-202103240746-0.
